# Association between 24-hour blood pressure parameters and 90-day functional outcome in acute ischemic stroke patients with early anticoagulation

**DOI:** 10.1097/MD.0000000000039181

**Published:** 2024-08-09

**Authors:** Lan Hu, Donggan Jin, Zhenguo Qiao, Wenze Hu, Yuan Xu, Yun Shi

**Affiliations:** a Department of Neurology, Suzhou Ninth People’s Hospital, Suzhou Ninth Hospital Affiliated to Soochow University, Suzhou, China; b Department of Neurology, Pujiang County People’s Hospital, Jinhua, China; c Department of Gastroenterology, Suzhou Ninth People’s Hospital, Suzhou Ninth Hospital Affiliated to Soochow University, Suzhou, China; d Department of Nursing, Ezhou Polytechnic, Ezhou, China; e Department of Obstetrics, Suzhou Ninth People’s Hospital, Suzhou Ninth Hospital Affiliated to Soochow University, Suzhou, China.

**Keywords:** acute ischemic stroke, blood pressure, blood pressure variability, outcome, stroke

## Abstract

This study aimed to examine the relationship between blood pressure (BP) and blood pressure variability (BPV) during the first 24 hours from admission with 90-day functional outcomes in acute ischemic stroke (AIS) patients whose onset within 24 hours and receiving early argatroban treatment. The study recruited 214 AIS patients. BP was monitored using a cuff at 1-hour fixed intervals, and BP/BPV parameters [standard deviation (SD), coefficient of variation (CV), successive variation (SV), and average real variability (ARV)] were collected. Age, the National Institutes of Health Stroke Scale (NIHSS) score at admission, previous history of diabetes mellitus (DM), and infarction site (located in anterior circulation) were identified as independent factors affecting 90-day outcomes in multiple logistic regression. After adjusting for confounding variables, association between BP/BPV and 90-day modified Rankin Scale (mRS) was assessed using logistic regression models. In model 1 (adjusted for age and NIHSS score at admission), mean-systolic blood pressure (SBP) showed association with 90-day outcomes [1.068 (1.008, 1.131), *P* = .025]. In model 2 (adjusted for age, NIHSS score at admission, previous history of DM), mean-SBP [1.061 (1.001, 1.123), *P* = .045] and max-SBP [0.951 (0.906, 0.998), *P* = .040] showed relatively weak association with outcomes. In model 3 [adjusted for age, NIHSS score at admission, previous history of DM, infarct site (located in anterior circulation)], all BP values were not related with outcomes, meanwhile, none of the BPV parameters calculated from SBP, diastolic blood pressure and mean arterial pressure showed association with 90-day outcomes. Future prospective studies are required to assess the relationship between early BP/BPV parameters with 90-day outcomes and further clarify the reference values for BP parameters. This is important for effective BP/BPV management and improved patient prognosis.

## 1. Introduction

More studies have explored the association between blood pressure (BP)^[1–4]^ and BP variability (BPV)^[5–9]^ with prognosis in acute ischemic stroke (AIS) patients. So far, the management of BP in acute phase remains confusing, and the results of studies focused on influence of BP/BPV on prognosis are inconsistent, which means well-designed studies on BP/BPV and outcomes are urgently needed. BP index plays a more important role in patients treated with special strategy in acute stage of stroke, and specific high BP thresholds are given for thrombolysis and thrombectomy patients.^[10,11]^ Compared with antiplatelet therapy, anticoagulation, intravenous thrombolysis, arterial thrombectomy or bridging strategies are classified as specialized or individual therapy. Nowadays, the most proportion of studies have focused on the association between BP/BPV and prognosis in patients receiving intravenous thrombolysis^[12,13]^ and arterial thrombolysis.^[14–16]^ It must be admitted that, only a tiny proportion of patients can receive these treatments. The application of new anticoagulant drugs in acute phase of AIS patients has gradually been recognized by clinicians, the most concern of this treatment strategy is the risk of bleeding and cerebral hyperperfusion, in fact, BP/BPV in this population should be monitored as closely as in patients with thrombolytic or thrombectomy, but, on the contrary, there has been long-term lack of clinical attention on BP/BPV in undergoing anticoagulation group. This work aim to explore the association between BP/BPV (initial 24 hours after admission) with 90-day functional outcomes in AIS patients treated with intravenous argatroban within 24 hours from stroke onset.

## 2. Methods

### 2.1. Subjects

Patients with AIS, who had received intravenous argatroban therapy within 24 hours from symptom onset were consecutively recruited between January 2016 and February 2020 from Suzhou Ninth Hospital affiliated with Soochow University. This study was approved by the Ethics Committee of Suzhou Ninth People’s Hospital and conducted in accordance with the declaration of Helsinki. Written informed consent was obtained from all participants.

The study inclusion criteria included: age > 18 years; new responsible infarction confirmed by cranial computed tomography (CT) or diffusion-weighted imaging (DWI) following AIS diagnostic criteria^[17]^; symptom onset within 24 hours and intravenous argatroban administered during initial 24-hour period; comprehensive 24-hour continuous BP monitoring data, with hourly BP recordings; written informed consent obtained from patients or their immediate family members.

On the other hand, the exclusion criteria were included: undergone intravenous thrombolysis, thrombectomy, or bridging treatment after stroke; BP interventions are required during hospitalization; unable to undergo cranial CT or DWI; severe liver or kidney diseases; history of blood diseases or illnesses with a bleeding tendency; history of malignant tumors or currently receiving anti therapy; currently pregnant and breastfeeding, or those planning to conceive; participated or were currently participating in other interventional clinical trials within the past 3 months; unsuitable for participation in this study considered by researchers.

A total of 214 patients who met the study criteria were recruited into the study.

### 2.2. Research methods

This study is a retrospective observational analysis. We collected demographic characteristics, vascular risk factor profiles, clinical variables, and imaging data of the included subjects. BP was measured in the nonparalytic upper arm using an electronic cuff at 1-hour interval (the time of admission was the first BP time), with continuous monitoring for 24 hours, resulting in 24 BP data points for each patient (IMEC 12 monitor; Mindray, Shenzhen Mindray Bioelectronics Co., Ltd., China).

Patients were divided into 2 groups based on modified Rankin Scale (mRS) at 90 days with good (mRS score ≤ 2) and poor outcome (mRS score > 2).

### 2.3. Definition of BPV

(1) Standard deviation of mean BP (SD):


$$\begin{array}{l} SD=\sqrt{1/\left(n-1\right)\overset{n-1}{\underset{i=1}{\sum }}\left(BP_{i}-BP_{mean}\right)} \end{array}$$

(2) Coefficient of variability (CV [%]):


$$\begin{array}{l} CV=\frac{SD}{BP_{mean}}\times 100 \end{array}$$

(3) Successive variation (SV):


$$\begin{array}{l} SV=\sqrt{1/\left(n-1\right)\overset{n-1}{\underset{i=1}{\sum }}{\left(BP_{I+1}-BP_{I}\right)}^{2}} \end{array}$$

(4) Average real variability (ARV):


$$\begin{array}{l} ARV=\frac{1}{n-1}\overset{n-1}{\underset{i=1}{\sum }}\left|BP_{i-1}-BP_{i}\right| \end{array}$$

### 2.4. Intravenous anticoagulation program

Argatroban (Tianjin Institute of Pharmaceutical Research Co., Ltd., China; 10 mg/dose, total of 60 mg) was intravenously administered for 2 consecutive days, followed by 10 mg via intravenous drip twice daily for 5 days. The total treatment duration was 7 days. The time interval between patient admission and intravenous administration of argatroban was within 1 hour.

### 2.5. Statistical analysis

Statistical analysis was performed using R version 4.0.2. Multiple imputations of missing continuous variables were conducted using multivariate imputation by chained equations. Baseline data were compared between groups using univariate logistic regression, and variables with *P* < .05 were included in the multivariate logistic models for adjustment. In the univariate analysis, BP and BPV parameters were compared between good and poor outcome groups using Wilcoxon rank-sum test. Variables with *P* < .2 from the univariate analysis were included in subsequent adjusted models. Model 1 used age and NIHSS score at admission as control variables, and the association of BP/BPV with 90-day outcomes were evaluated. Based on model 1, history of DM as a control variable was added into model 2, and the impact of BP/BPV on prognosis was assessed again. Furtherly, based on model 2, the influence of BP/BPV parameters on 90-day outcomes were assessed in model 3 by adding infarction site (located in anterior circulation) as a control variable.

## 3. Results

### 3.1. Baseline characteristics

Among the 214 patients (mean age: 68.02 ± 11.91 years; female: 37.4%; NIHSS score at admission: 5^[3,8]^), 123 patients (57.48%) had good outcome (mRS score ≤ 2; see Appendix Table 1, http://links.lww.com/MD/N317 for baseline characteristics). Age (*P* = .001), NIHSS score at admission (*P* < .001), history of DM (*P* = .011), and infarction site (*P* = .027) were associated with 90-day outcomes. In poor outcome group, age, NIHSS score at admission, the proportion of patients with DM and infarction located in anterior circulation were higher than that in good outcome group (*P* < .05). Other factors showed no significant difference between the 2 groups (Table 1). Multiple logistic regression revealed age (odds ratio [OR] = 1.020, 95% confidence interval [CI] 0.992–1.049, *P* = .165), NIHSS score at admission (OR = 1.302, 95% CI 1.175–1.464, *P* < .001), history of DM (OR = 2.583, 95% CI 1.348–5.080, *P* = .005), and infarction site (located in anterior circulation; OR = 2.309, 95% CI 1.167–4.670, *P* = .018) as independent impact factors of 90-day functional outcomes.

**Table 1:** Univariate and multivariate analysis of the correlation between baseline data and 3-month prognosis in acute ischemic stroke patients receiving early anticoagulation.*

Variables	Mono factor analysis		Multiple factor analysis	
	OR (95% CI)	*P* value	OR (95% CI)	*P* value
Age (yr, $\bar{x}\pm s$)	1.042 (1.017–1.068)	.001	1.020 (0.992–1.049)	.165
Female (%)	1.282 (0.731–2.265)	.388		
NIHSS score at admission ($\bar{x}\pm s$)	1.317 (1.196–1.470)	<.001	1.302 (1.175–1.464)	<.001
Platelet volume (fL, $\bar{x}\pm s$)	1.042 (0.946–1.161)	.424		
BNP (pg/mL, $\bar{x}\pm s$)	1.000 (0.999–1.001)	.651		
Platelet counting (×10^9^/L, $\bar{x}\pm s$)	0.999 (0.994–1.003)	.533		
CTn-I > 0.04 (ng/mL)	1.689 (0.591–5.527)	.347		
Scr (µmol/L, $\bar{x}\pm s$)	1.003 (0.992–1.014)	.640		
Plasma D–D polymers (ng/mL, $\bar{x}\pm s$)	1.059 (0.818–1.427)	.673		
FIB (g/L, $\bar{x}\pm s$)	0.972 (NA–1.022)	.445		
CRP (mg/dL, $\bar{x}\pm s$)	1.025 (1.003–1.059)	.084		
HbA1c (%, $\bar{x}\pm s$)	1.065 (0.906–1.259)	.447		
Albumin (g/L, $\bar{x}\pm s$)	1.000 (0.972–1.028)	.973		
LDL (mmol/L, $\bar{x}\pm s$)	1.111 (0.808–1.535)	.518		
Hcy (µmol/L, $\bar{x}\pm s$)	0.991 (0.968–1.014)	.440		
Hypertension [n (%)]	1.756 (0.862–3.618)	.122		
Diabetes mellitus [n (%)]	2.074 (1.182–3.695)	.011	2.583 (1.348–5.080)	.005
Anterior circulation infarction [n (%)]	1.937 (1.077–3.512)	.027	2.309 (1.167–4.670)	.018

BNP = brain natriuretic peptide, CI = confidence interval, CRP = C-reactive protein, CTn-I = cardiac troponin I, FIB = fibrinogen, HbA1c = glycosylated hemoglobin A1c, Hcy = homocysteine, LDL = low-density lipoprotein cholesterol, NA = not applicable, NIHSS = National Institutes of Health Stroke Scale, OR = odds ratio, Scr = serum creatinine.

* All continuous variables with missing values were imputed by multivariate imputation by chained equations, and logistic regression was used for mono and multiple factor analysis after imputation, mono and multiple factor analysis data sets are consistent.

### 3.2. Comparison of BP and BPV-related parameters in good and poor outcome groups

Table 2 presents the comparison of BP/BPV parameters between groups. Among these parameters, initial systolic BP (initial-SBP; *P* = .077), mean systolic BP (mean-SBP; *P* < .001), maximum systolic blood pressure (max-SBP; *P* = .013), minimum systolic blood pressure (min-SBP; *P* = .017), systolic blood pressure standard deviation (SBP-SD; *P* = .081), systolic blood pressure coefficient of variation (SBP-CV; *P* = .060), mean arterial pressure (MAP; *P* = .017), maximum MAP (*P* = .067), and minimum MAP (*P* = .020; *P* < .2) were brought into the subsequent models for analysis. Moreover, the values of these parameters were higher in good outcome group than that in the poor outcome group.

**Table 2:** Univariate analysis of BP and BPV-related parameters in AIS patients with good and poor outcomes.

BP parameters	Total (n = 214)	mRS ≤ 2 (n = 123)	mRS > 2 (n = 91)	*P* value
SBP				
initial	160.43 ± 26.28	156.75 ± 24.94	163.16 ± 27.01	.077
mean	143.83 ± 17.27	138.79 ± 15.73	147.56 ± 17.47	<.001
max	172.18 ± 21.67	167.93 ± 20.02	175.33 ± 22.38	.013
min	116.96 ± 16.11	113.91 ± 13.96	119.22 ± 17.24	.017
SD	13.69 ± 4.51	13.06 ± 3.68	14.15 ± 4.99	.081
CV	13.71 ± 4.35	13.05 ± 3.62	14.19 ± 4.78	.060
SV	3.09 ± 0.84	3.07 ± 0.81	3.11 ± 0.87	.696
ARV	11.39 ± 2.95	11.25 ± 2.82	11.50 ± 3.06	.541
DBP				
initial	88.24 ± 14.27	89.40 ± 13.25	87.38 ± 14.98	.309
mean	80.99 ± 10.93	80.24 ± 10.44	81.54 ± 11.28	.393
max	99.57 ± 12.10	98.57 ± 11.33	100.32 ± 12.63	.298
min	62.35 ± 10.54	61.41 ± 9.72	63.04 ± 11.10	.263
SD	9.29 ± 2.56	9.23 ± 2.38	9.33 ± 2.70	.784
CV	9.16 ± 2.41	9.14 ± 2.28	9.18 ± 2.52	.914
SV	2.32 ± 0.64	2.30 ± 0.62	2.35 ± 0.67	.600
ARV	8.61 ± 2.39	8.43 ± 2.28	8.75 ± 2.48	.348
MAP				
mean	101.94 ± 11.68	99.73 ± 10.90	103.58 ± 12.00	.017
max	121.43 ± 13.86	119.41 ± 12.84	122.92 ± 14.44	.067
min	82.76 ± 11.39	80.67 ± 10.28	84.32 ± 11.95	.020
SD	9.60 ± 2.89	9.49 ± 2.46	9.96 ± 3.18	.619
CV	9.47 ± 2.74	9.55 ± 2.40	9.42 ± 2.98	.726
SV	2.23 ± 0.58	2.22 ± 0.55	2.23 ± 0.60	.952
ARV	8.31 ± 2.17	8.27 ± 2.15	8.34 ± 2.19	.801

ARV = average real variability, BP = blood pressure, CV = coefficient of variation, DBP = diastolic blood pressure, MAP = mean arterial pressure, max = maximum, min = minimum, mRS = modified Rankin Scale, SBP = systolic blood pressure, SD = standard deviation, SV = successive variation.

### 3.3. Multivariable logistic regression model adjusted for potential confounding factors to access the impact of BP and BPV-related parameters on 90-day outcomes

BP/BPV parameters with *P* < .2 were incorporated into the adjusted multivariable logistic regression model (Table 3). In model 1 (adjusted for age and NIHSS score at admission), mean-SBP exhibited association to a certain extent with 90-day outcomes (OR = 1.068, 95% CI 1.008–1.131, *P* = .025). In model 2 (based on model 1, adding history of DM as a variable), both mean-SBP (OR = 1.061, 95% CI, 1.001–1.123, *P* = .045) and max-SBP (OR = 0.951, 95% CI, 0.906–0.998, *P* = .040) demonstrated a modest relationship with 90-day outcomes; In model 3 (based on model 2, infarction site as a variable was added), all BP values were not related with outcomes, as the number of variables increased, the correlative OR decreased gradually. Meanwhile, none of the BPV parameters calculated from SBP, diastolic blood pressure (DBP) and MAP showed association with 90-day outcomes.

**Table 3:** Multivariable logistic regression model adjusting for confounding factors to assess the impact of BP and BPV-related parameters on 90-day outcomes.

BP/BPV parameters	Model 1		Model 2		Model 3	
	OR (95% CI)	*P* value	OR (95% CI)	*P* value	*OR* (95% CI)	*P* value
initial-SBP	1.009 (0.997–1.021)	.147	1.009 (0.997–1.022)	.142	1.009 (0.997–1.022)	.133
mean-SBP	1.068 (1.008–1.131)	.025	1.061 (1.001–1.123)	.045	1.057 (0.998–1.120)	.059
max-SBP	0.957 (0.912–1.003)	.067	0.951 (0.906–0.998)	.040	0.952 (0.906–1.000)	.049
min-SBP	1.007 (0.958–1.058)	.793	1.018 (0.966–1.072)	.506	1.019 (0.966–1.074)	.490
SD-SBP	0.999 (0.640–1.557)	.995	1.017 (0.647–1.598)	.943	1.079 (0.677–1.720)	.749
CV-SBP	1.153 (0.741–1.793)	.528	1.175 (0.750–1.840)	.482	1.117 (0.706–1.768)	.637
mean-MAP	1.059 (0.970–1.156)	.201	1.047 (0.957–1.145)	.317	1.036 (0.946–1.135)	.450
max-MAP	0.982 (0.935–1.033)	.489	0.988 (0.938–1.039)	.630	0.994 (0.944–1.047)	.831
min-MAP	1.002 (0.948–1.059)	.943	1.007 (0.951–1.066)	.815	1.009 (0.953–1.069)	.750

Model 1: adjusted age and NIHSS at admission; Model 2: adjusted age, NIHSS score at admission and diabetes mellitus; Model 3: adjusted age, NIHSS at admission, diabetes mellitus and anterior circulation.

ARV = average real variability, BP = blood pressure, BPV = blood pressure variability, CI = confidence interval, CV = coefficient of variation, DBP = diastolic blood pressure, MAP = mean arterial pressure, max = maximum, min = minimum, mRS = modified Rankin Scale, OR = odds ratio, SBP = systolic blood pressure, SD = standard deviation, SV = successive variation.

## 4. Discussion

Ischemic stroke (IS) presents a major health risk, with high mortality and disability, putting a substantial health burden on society and families.^[18,19]^ Intravenous thrombolysis and intra-arterial therapy have been shown to improve the prognosis of AIS patients and are highly recommended by clinical guidelines.^[20,21]^ Nonetheless, the above treatments are limited by time/tissue window, and high cost of treatment, currently only can implemented in a very small proportion of stroke patients. In the real world, early anticoagulation acts as an important intervention for AIS therapy.^[22–25]^ Argatroban, a direct thrombin inhibitor, rapidly inhibits thrombosis, and its safety and efficacy in AIS patients have been confirmed by many clinical studies.^[26–30]^

In some studies, BPV exhibited strong association with stroke prognosis,^[31,32]^ especially SBP variability indicators in the first 24-hour from onset.^[33,34]^ Initial 24-hour systolic BPV in AIS patients is a strong predictor of stroke outcomes, with higher systolic BPV indicating a worse prognosis.^[8,9]^ In line with VISTA study, not only SBP-SD was shown to have a statistically significant impact on prognosis at 90 days, but also DBP-SD and MAP-SD, high 24-hour SBP-SD, DBP-SD, and MAP-SD were related to 90-day adverse outcomes (mRS score of 3–6).^[35]^ In addition, exception for SD, CV also brought out prognostic relevance, increased SBP-CV and DBP-CV in AIS patients were associated with higher risk for unfavorable outcome at 3 months independent of BP levels.^[32]^

However, there were differences between our findings and these studies, our research suggested that SBP, DBP, and MAP related indices (SD, CV, SV, and ARV) were not related with 90-day functional outcomes, the conclusion was consistent with those of Graff and the Samurai rt-PA registration studies.^[36,37]^

Several possible factors may explain for the conflicting research findings. Firstly, the effect of BPV on prognosis may be mediated by vessel recanalization. Delgado-Mederos et al^[38]^ found that the effect of BPV on lesion enlargement and 90-day outcomes in AIS patients undergoing rt-PA thrombolysis was dependent on blood vessel recanalization. In poor recanalization group, systolic BPV acted as an independent predictor of infarction enlargement and poor prognosis. However, this predictive value disappeared in patients with successful recanalization. Several studies had demonstrated that BPV significantly affected the prognosis of AIS patients with carotid artery or intracranial large vessel stenosis or occlusion.^[13,39]^ Regrettably, this study did not radiographically assess recanalization and carotid artery or intracranial large vessel stenosis or occlusion in all patients. In the present study, 214 AIS patients received argatroban within 24 hours from onset, clinical symptoms of most patients relieved rapidly, none of them experienced symptomatic cerebral hemorrhage or systemic hemorrhage, thus, we speculated that early anticoagulant therapy may have an improvement effect on recanalization and do not increase the risk of cerebral hyperperfusion, through suppressing thrombus enlargement, dissolve partial thrombi, and promote their escape to the distal end of the vessel, thereby improving cerebral perfusion, reducing brain edema, and stabilizing blood pressure, which would help decrease BPV, ultimately leading no significant effect on 90-day prognosis. In addition, this population had a median NIHSS score of 5 points on admission, combined with this baseline NIHSS score at admission, we conjectured that the proportion with large artery disease maybe was relatively low, which may also be another important reason for the negative result.

Secondly, healthy individuals possess autonomic function, which is impaired after AIS. Previous studies indicated that significant autonomic dysfunction was responsible for poor prognosis. Researchers speculated that this dysfunction may cause BP fluctuations during the acute phase, potentially affecting prognosis.^[40]^ Building on this, we furtherly inferred that remarkable BP fluctuations after significant autonomic dysfunction could increase BPV. The more pronounced the autonomic dysfunction, the greater effect on BPV parameters consequently resulted in functional prognosis. Notably, patients in our study had a median NIHSS score of 5 points on admission, which means their neurological deficits were relatively mild, from this, we supposed the autonomic dysfunction was less impaired, therefore, had a limited influence on BPV.

Furthermore, studies have shown that AIS patients experience acute neurogenic stress, excessive catecholamine release from sympathetic nerves into myocardial cells and systemic circulation, and increased sensitivity to norepinephrine^[41]^; all of which can promote BP fluctuations and changes in BPV during the acute phase. Besides, stress disorders can induce poor functional prognosis for stroke patients.^[42]^ Consequently, it is plausible to infer that early neurogenic stress in AIS patients may cause greater BPV variation, thereby impacting prognosis. However, stress response is also associated with disease severity. A shift to sympathetic predominance has been previously linked to proinflammatory cytokine production, hyperglycemia, and increased blood-brain barrier permeability.^[43,44]^ Herein, the conditions of patients were relatively mild, and the ultra-early stress reaction may not have been severe following the NIHSS scores at admission and C-reactive protein levels, thereby minimally affecting BPV. This maybe be one of the reasons for the insufficiency of influence on 90-day prognosis.

Different studies use different methods of measuring BP and a study considered that different BP detection methods could yield varying results.^[3]^ Unlike Graff’s beat-to-beat finger BP detection method,^[36]^ our study and Samurai rt-PA registration study^[37]^ adopted cuff BP detection method. Although BP detection methods used in our study and Samurai rt-PA registration study, are completely different from which used in GRAff’s study, but the results consistently showed 24-hour SD, CV, SV, and ARV have no relationship with 90-day outcomes in AIS patients,^[36,37]^ which may be due to the fact that BPV is indeed not associated with prognosis and may not be associated with measurement method. Of course, the results of the study need to be interpreted with caution. After all, the study population in this study was those who received intravenous argatroban anticoagulation and had an average NIHSS score of 5 at admission, while the enrolled population in the above 2 studies were all AIS patients, and there were certain differences in the study population.

Although the SD, CV, SV and ARV derived from SBP in this study did not show a significant relationship with 90-day outcomes, a slight correlation was observed between max-SBP with 90-day outcomes in model 1 and model 2. According to the INSPIRE study,^[45]^ increased baseline BP was associated with better collateral flow, in subgroup analysis of patients with major reperfusion, higher BP was associated with decreased infarct growth and a better clinical outcome. Therefore, it is reasonable to speculate that max-SBP may affect functional prognosis by collateral circulation and cerebral perfusion. The effect of max-SBP on prognosis should be further investigated in subsequent studies. Meanwhile, in this study, DBP indicators (mean-DBP, max-DBP, min-DBP) and MAP-related indicators showed no association with 90-day outcomes. This may be due to the advanced age of AIS patients, SBP has a greater impact on cerebral perfusion than DBP and MAP, once stroke occurs, the self-regulation mechanism of cerebral blood flow is affected, and the survival of the ischemic penumbra is more significantly influenced by systemic SBP.

Currently available guidelines do not provide specific recommendations for BP management in the ultra-early stage for BP levels below 220/120 mm Hg.^[20]^ Active and rapid reduction of BP is not recommended unless specific reasons. This recommendation is based on considerations related to the self-regulation mechanism of the brain, cerebral perfusion, and the potential impact of BPV on prognosis. Nonetheless, the relationship between BP/BPV and stroke prognosis remains unknown, making research in this area particularly meaningful, particularly for AIS patients under specialized treatments. Moreover, limited studies previously focused on the prognostic effect of BP/BPV on the prognosis of AIS patients receiving early anticoagulation therapy. As such, our research findings can provide a reference for clinicians, and additional studies in the future should focus on the effect of BP/BPV on the prognosis of AIS patients undergoing early anticoagulation.

This study has compelling limitations that warrant consideration. First, we adopted a retrospective design and a small sample size. Secondly, we employed an electronic cuff for BP monitoring, which differs from more precise methods described in the literature, including beat-to-beat BP monitoring. Thirdly, we did not account for the effects of heart rate on BP, and BPV was monitored for only 24 hours, rather than for 48 to 72 hours or 1 week. Furthermore, BP fluctuations in patients with AIS were prominent during the initial hours, yet we did not comprehensively analyze BPV within the first 0 to 6 hours, nor did we differentiate between diurnal and nocturnal BPV in relation to 90-day outcomes. We also underestimated the infarct size, intracranial and extracranial vascular conditions in AIS patients, and stroke subtypes remained undifferentiated. The focus of our future research will be assessing the relationship between BP/BPV and outcomes, which may be more accurate when incorporating relevant imaging examinations and stroke subtypes. In addition, additional studies are necessary to investigate the relationship between BP/BPV and outcomes in patients receiving early intravenous argatroban therapy.

## 5. Conclusion

In conclusion, the first 24 hours BP/BPV parameters (SD/CV/SV/ARV) after admission demonstrated no significant association with 90-day functional outcomes in AIS patients receiving argatroban treatment within 24 hours from stroke onset. Therefore, future prospective studies are necessary to assess the relationship between early BP and BPV parameters with 90-day outcomes and further clarify the reference values for BP/BPV parameters. This is necessary for effective BP management and improved patient outcomes.

## Author contributions

**Conceptualization:** Yun Shi.

**Data curation:** Donggan Jin.

**Formal analysis:** Wenze Hu.

**Funding acquisition:** Yuan Xu.

**Investigation:** Donggan Jin.

**Methodology:** Yun Shi.

**Writing – original draft:** Lan Hu.

**Writing – review & editing:** Zhenguo Qiao.

## Supplementary Material
