# Supplementary material for: Association between 24-hour blood pressure parameters and 90-day functional outcome in acute ischemic stroke patients with early anticoagulation
Source: Medicine (Baltimore). 2024 Aug 9;103(32):e39181. doi: 10.1097/MD.0000000000039181 (PMC11315527; doi:10.1097/MD.0000000000039181)
Supplement: Supplementary file 1 [file medi-103-e39181-s001.docx]

**Appendix**

**Table S1 -** Comparison of baseline characteristics in patients with AIS in the good/poor outcome group.

|  | Total (n = 214) | mRS≤2 (n = 123) | mRS>2 (n = 91) | *P* value |
| --- | --- | --- | --- | --- |
| Age (y, $\bar{x}$±*s*) | 68.02±11.91 | 64.81±11.61 | 70.40±11.62 | 0.001 |
| Female (%) | 80 (37.4) | 31 (14.5) | 49 (22.9) | 0.388 |
| NIHSS score at admission (*M* [IQR]) | 5 (3, 8) | 3 (2, 5) | 7 (4, 10) | <0.001 |
| Platelet volume (fl, $\bar{x}$±*s*) | 10.46±2.75 | 10.06±2.18 | 10.72±3.04 | 0.132 |
| BNP (pg/ml, $\bar{x}$±*s*) | 209.02±321.72 | 184.21±314.94 | 222.55±326.34 | 0.509 |
| Platelet counting (×10^9^/L, $\bar{x}$±*s*) | 192.30±61.76 | 202±56.07 | 186.32±59.19 | 0.129 |
| CTn-I (ng/ml, $\bar{x}$±*s*) | 0.11±0.77 | 0.01±0.01 | 0.18±1.01 | 0.151 |
| Scr (µmol/L, $\bar{x}$±*s*) | 68.26±25.88 | 67.30±27.8 | 68.98±24.45 | 0.640 |
| Plasma D-D polymers (ng/ml, $\bar{x}$±*s*) | 0.62±1.09 | 0.58±0.99 | 0.66±1.15 | 0.580 |
| FIB (g/L, $\bar{x}$±*s*) | 3.53±8.30 | 4.53±13.50 | 2.92±0.65 | 0.290 |
| CRP (mg/dl, $\bar{x}$±*s*) | 8.95±19.15 | 5.53±7.19 | 11.20±23.76 | 0.062 |
| HbA1c (%,$\bar{x}$±*s*) | 6.55±1.69 | 6.39±1.57 | 6.66±1.76 | 0.382 |
| Albumin (g/L, $\bar{x}$±*s*) | 36.63±10.16 | 36.77±11.08 | 36.54±9.55 | 0.882 |
| LDL (mmol/L, $\bar{x}$±*s*) | 2.74±0.85 | 2.69±0.85 | 2.78±0.85 | 0.451 |
| Hcy (µmol/L, $\bar{x}$±*s*) | 15.61±11.92 | 16.67±13.83 | 14.73±10.05 | 0.280 |
| Hypertension [n (%)] | 177 (82.7) | 71 (33.2) | 106 (49.5) | 0.119 |
| Diabetes mellitus [n (%)] | 87 (40.7) | 28 (13.1) | 59 (27.6) | 0.011 |
| Anterior circulation infarction [n (%)] | 149 (69.6) | 56 (26.2) | 93 (43.4) | 0.027 |

**Abbreviations**: NIHSS, National Institutes of Health Stroke Scale; BNP, brain natriuretic peptide; CTn-I, Troponin; Scr, serum creatinine; FIB, fibrinogen; CRP, C-reactive protein; HbA1c, glycosylated hemoglobin A1c; LDL, low-density lipoprotein cholesterol; Hcy, homocysteine; NA: not applicable
